# A detailed account of the measurements of cold collisions in a molecular synchrotron

**DOI:** 10.1140/epjti/s40485-018-0048-y

**Published:** 2018-07-11

**Authors:** Aernout P. P. van der Poel, Hendrick L. Bethlem

**Affiliations:** LaserLaB, Department of Physics and Astronomy, Vrije Universiteit, De Boelelaan 1081, Amsterdam, The Netherlands

**Keywords:** Cold molecules, Stark deceleration, Collisions, Molecular beams

## Abstract

We have recently demonstrated a general and sensitive method to study low energy collisions that exploits the unique properties of a molecular synchrotron (Van der Poel et al., Phys Rev Lett 120:033402, 2018). In that work, the total cross section for ND3 + Ar collisions was determined from the rate at which ammonia molecules were lost from the synchrotron due to collisions with argon atoms in supersonic beams. This paper provides further details on the experiment. In particular, we derive the model that was used to extract the relative cross section from the loss rate, and present measurements to characterize the spatial and velocity distributions of the stored ammonia molecules and the supersonic argon beams.

## Review

Collision studies at low temperatures are of interest from both a practical and theoretical viewpoint. Interstellar clouds, which make out a large fraction of our universe, typically have temperatures well below 100 K. Collision data of simple molecules at low temperatures is crucial for understanding the chemistry that goes on in these clouds, which is of special interest because it is from these clouds that solar systems form [1, 2]. Furthermore, at low temperatures the de Broglie wavelength, associated with the relative velocity of the colliding molecules, becomes comparable to, or larger than, the intermolecular distances and quantum effects become important. Particularly interesting are resonances of the collision cross section as a function of collision energy. The position and shape of these resonances are very sensitive to the exact shape of the potential energy surface (PES) and thus serve as precise tests of our understanding of intermolecular forces [3–6]. Precise knowledge of the PES is fundamental to fields such as combustion physics, atmospheric physics, or in fact any field involving chemical reactions.

Although several techniques have been developed to create samples of cold molecules [7–10], the obtained densities are low (typically 10^8^ molecules/cm^3^). As the cross sections of collisions involving neutral molecules or atoms are small (typically below 500 Å^2^), the main challenge to studying cold collisions is to reach a sufficiently high sensitivity. In recent years, several experiments have managed to measure low energy collisions by leveraging the unique properties of the systems they study. For instance, by exploiting the extreme state-purity of Stark-decelerated beams combined with sensitive ion-detection techniques, van de Meerakker and co-workers have measured quantum-state changing collisions of OH and NO molecules with rare gas atoms to temperatures as low as 5 K [11–14]. Costes and co-workers have studied inelastic collisions of O_2_ and CO with H_2_ molecules and helium at energies between 5 and 30 K using cryogenically cooled beams under a small (and variable) crossing angle [15–17]. Even lower temperatures have been obtained by using magnetic or electric guides to merge two molecular beams. Narevicius and co-workers and Osterwalder and co-workers have exploited the advantages of metastable helium to study Penning ionization reactions with various atoms and molecules [18–26]. In a similar fashion, Merkt and co-workers have measured collisions between ground-state hydrogen molecules and hydrogen in highly excited Rydberg states that were merged on a chip [27]. Finally, cold collision have been studied by sending slow beams of atoms and molecules through trapped samples of calcium ions [28, 29], lithium atoms [30] and OH radicals [31], exploiting the fact that collision signal can be accumulated over long time-intervals.

We have developed a method that enables the study of collisions at low energy by exploiting the unique properties of a molecular synchrotron. Our approach combines the low collision energies obtained in experiments that use merged molecular beams with the high sensitivity of experiments that monitor trap loss. In Van der Poel et al. [32], the total cross section for ND_3_ + Ar collisions was determined from the rate at which ammonia molecules were lost from the synchrotron due to collisions with argon atoms in supersonic beams. This paper provides further details on this experiment. Before going into detail, we will first outline the main principles and virtues of our technique.

## Main principles

In its simplest form, a storage ring is a trap that confines molecules along a circle rather than around a point. As such, a storage ring for molecules in low-field seeking states can be made by bending an electrostatic hexapole focuser into a circle, which was demonstrated in 2001 by Crompvoets et al. [33]. Since in such a storage ring no longitudinal forces exist to keep the faster and slower molecules together, injected packets of molecules will disperse until the ring is filled homogeneously. As demonstrated in 2007 by Heiner et al. [34], this can be prevented by breaking the ring into two half-circles and switch the voltages in such a way that molecules are bunched together as they fly through the gap between the two half rings. In 2010, an improved synchrotron, consisting of 40 straight hexapoles placed in a circle was demonstrated by Zieger et al. [35]. Using many segments rather than two has a number of advantages: Due to the high symmetry ring of the, instabilities resulting from the variation of the trapping force are less important and the transverse depth of the ring is increased. The longitudinal well is also increased as bunching happens many times per round-trip. Zieger et al. illustrated the stability of their design by demonstrating that they could observe trapped ammonia molecules (ND_3_) in well-defined, mm-sized packets, after storing them for more than 10 s in the ring [35]. The fact that elements can be switched individually implies that different packets can be injected and detected independently, allowing the synchrotron to hold up to 19 packets simultaneously. As the stored packets of ammonia molecules have both a small velocity spread (corresponding to a temperature of ∼10 mK) and widely (100–150 m/s) tunable velocities, they are well suited for collision studies, as will be demonstrated in this paper.

**Fig. 1:**
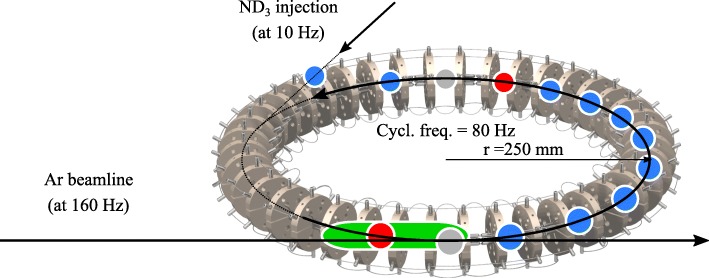
Schematic sketch of the synchrotron and argon beamline. The red circles denote probe packets of ammonia molecules which are targeted by the argon beam (shown in green). The blue circles denote reference packets of ammonia, which do not encounter argon atoms and provide a simultaneous background measurement. The grey circles denote reference packets of ammonia that encounter the leading or trailing end of the argon beams, and are therefore discarded

The main idea of our experiment is illustrated in Fig. 1. Beams of argon atoms are sent through the synchrotron and made to collide with the stored packets of ammonia. The argon beams moves in the same direction as the stored ammonia molecules such that low collision energies can be achieved [15–17, 36, 37]. The experiment is triggered in such a way that some of the ammonia packets—the *probe* packets—encounter a fresh argon beam every round-trip, while other packets—the *reference* packets—do not. When after a certain number of round-trips the packets are detected, the probe and reference signals are compared to find the rate at which ammonia molecules are lost from the synchrotron due to collisions with the argon beam. The longer the packets are stored before detection, the more molecules are lost from the probe beam. In this way, collision signal is accumulated and the sensitivity to measure collisions is strongly enhanced.

**Fig. 2:**
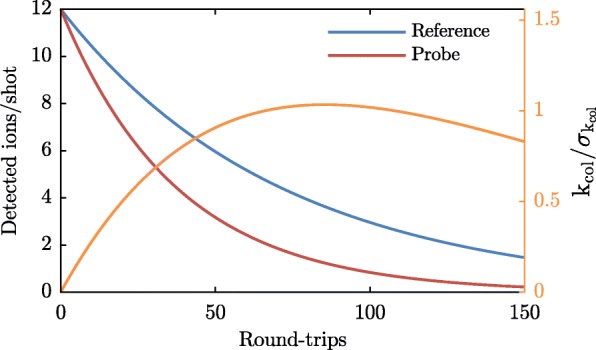
Model of the probe (red) and reference (blue) signals (using numbers from the experiment discussed in Van der Poel et al. [32]), and the signal-to-noise ratio (orange, right y-axis) that results when these signals are used to calculate the loss rate due to collisions *k*_col_ of the probe packets

The expected enhancement in sensitivity is illustrated in Fig. 2. The signals of the probe (red, *S*_probe_) and reference packets (blue, *S*_ref_), using numbers from the experiment that was discussed in Van der Poel et al. [32]., are shown as a function of storage time in the synchrotron. Both signals are modelled by exponential decays. While the probe and reference packets share the same background loss rate (in this calculation *k*_bg_=1.46% per round-trip), the probe packets are modelled to experience additional loss due to collisions with particles from the collision partner beamline (at a rate of *k*_col_=1.26% per round-trip). After a given number of round-trips *rt*, the loss rate due to collisions can be found using

1 $$  k_{\text{col}}=\frac{1}{\text{rt}}\ln\left(\frac{S_{\text{ref}}}{S_{\text{probe}}}\right).  $$

The uncertainty in the loss rate is found from the statistical uncertainties in the probe and reference signals. Since the number of detected ions follows Poisson statistics, the uncertainty is given by the square-root of this number:

2 $$  \sigma_{\mathrm{k_{col}}}=\frac{1}{\text{rt}}\sqrt{\left(\frac{1}{S_{\text{ref}}}\right)+\left(\frac{1}{S_{\text{probe}}}\right).}  $$

The orange curve shows the ratio of the calculated loss rate *k*_col_ and its uncertainty $\sigma _{k_{\text {col}}}$, after a single measurement only (i.e., a single measurement of a probe and a reference packet, requiring two shots). This signal-to-noise ratio increases dramatically the first tens of round-trips, due to the factor of 1/rt in $\sigma _{k_{\text {col}}}$. After about 90 round-trips, roughly 2 times the 1/*e* lifetime of the probe packet, the statistical uncertainty in *S*_probe_ becomes the limiting factor and the signal-to-noise ratio decreases again. For the numbers used in this calculation, the expected signal-to-noise ratio at the optimal number of round-trips is ∼1 after a single measurement of the probe packet and reference packet. The uncertainty can thus be reduced to below 1% by measuring 20,000 shots, or a little over half an hour when measuring at a rate of 10 Hz. Note that the signal-to-noise ratio at the optimal number of round-trips is 34 times larger than after a single round-trip. Consequently, the sensitivity of the synchrotron reduces the measuring time by over a factor of 1000 with respect to a hypothetical crossed beam experiment with the same densities. This enhancement in sensitivity is what motivated us to do this experiment.

Since the collision partners move in the same direction as the stored molecules, the collision energy is determined by the difference in velocity. Thus, by choosing beams that move with the same speed as the stored molecules, one can, in principle, measure collisions at zero collision energy. Unfortunately, our current synchrotron only allows us to store molecules with a speed up to 150 m/s, which is much lower than the speed of the argon beams used in our experiment (420-570 m/s). As a result of this, the lowest collision energy obtained in our work is 40 cm ^−1^ (corresponding to 56 K).

The rest of this paper is organized as follows: The first two sections provide a detailed overview and characterization of the experimental setup. We present measurements of the argon beams at two positions along the beamline to determine the position and velocity distributions of the argon beam at any position and time, and discuss the position and velocity distributions of the ammonia molecules inside the ring. In the succeeding section, we present a model that combines trajectory simulations of ammonia molecules in the synchrotron with the argon beam. This critical piece of the puzzle also provides the collision energy distributions probed in the experiments. In the succeeding section, all pieces are combined, and measurements are presented of the ND_3_ + Ar cross section as a function of collision energy. The paper ends with conclusions and a short discussion on possible future experiments.

## Molecular synchrotron

**Fig. 3:**
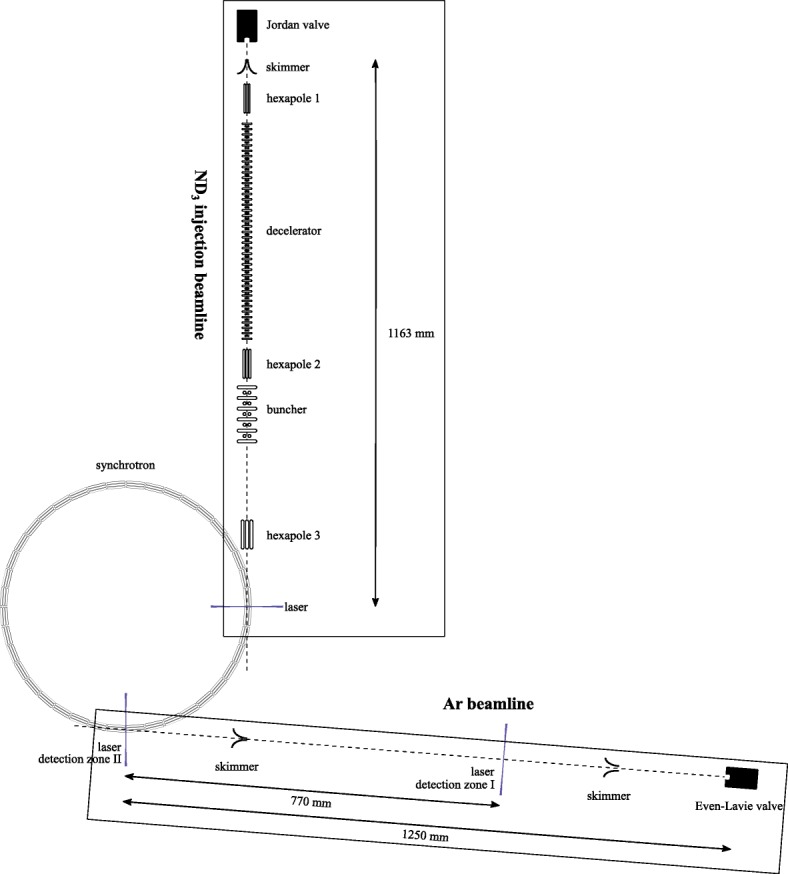
Top view of the experimental set-up (to scale). ND_3_ molecules are decelerated to velocities of 100–150 m/s using a Stark decelerator and injected into a molecular synchrotron consisting of 40 electric hexapole elements arranged in a half-meter-diameter circle. Ammonia molecules can be detected at the injection point or at a quarter ring downstream (detection zone II). The argon beamline consists of a cooled solenoid valve that is skimmed by a 5 mm and a 1.5 mm skimmer. Argon atoms are detected 770 mm in front of the synchrotron (detection zone I) or inside the synchrotron (detection zone II). The argon beamline makes an angle of 94,5 degrees with respect to the injection beamline such that the argon beam moves parallel to the first hexapole in the synchrotron behind detection zone II

Our molecular synchrotron, schematically depicted in Fig. 3, consists of 40 electric hexapole elements arranged in a half-meter-diameter circle. Voltages of up to ±5 kV are applied to the electrodes in order to transversely trap ammonia molecules in the low-field seeking sublevel of the *J*=1, *K*=1 rovibrational ground state within the hexapoles. The molecules are bunched longitudinally by switching the voltages temporarily to higher voltages as the molecules fly through the gap between one hexapole and the next. In principle 20 packets can be stored simultaneously, with velocities of 100–150 m/s. In the experiments presented here, we store 14 packets with a velocity of either 121.1 m/s or 138.8 m/s.

New packets are injected into the synchrotron by an injection beamline consisting of (1) a Gentry type pulsed valve (R.M. Jordan company), that releases packets of 5% ammonia seeded into xenon with velocities around 350 m/s, (2) a Stark decelerator, that decelerates ammonia molecules in the *J*=1, *K*=1 rovibrational ground state to velocities down to 100 m/s, (3) a buncher that focuses the molecules longitudinally into the synchrotron, and (4) hexapole elements that focus the molecules transversely. To allow the molecules to enter the synchrotron, 4 hexapole elements of the synchrotron are temporarily switched off. The beamline is synchronized with the cyclotron frequency of the stored molecules in such a way that new packets are injected two hexapole segments in front of the packets that are already stored, at a rate around 10 Hz. For molecules that are stored at a velocity of 121.1 m/s, this implies that they make 6+38/40 round-trips before a new packet is injected, while molecules stored at a velocity of 138.8 m/s make 7+38/40 round-trips before a new packet is injected.

The velocity of the injected ammonia molecules is determined by the trigger sequence applied to the Stark decelerator, and can be changed almost instantly; it takes 1.4 s to load the synchrotron with new packets after changing the cyclotron frequency and the trigger sequence. In this way, the collision energy can be varied on relatively short timescales to counteract possible drifts of the intensity and timing of the argon beam during collision measurements.

At the same rate that new packets are injected, the oldest packet in the synchrotron is detected at one of two detection zones: A focused laser pulse (typically 5 ns long, 10 mJ/pulse, 317 nm) ionizes ammonia molecules by 2+1 Resonance-Enhanced Multi-Photon Ionization (REMPI) *via* the electronic B-state. The UV-beams are focused in-between the hexapole elements using lenses with focal lengths of 50 mm, which are mounted on three-dimensional translation stages to allow precise scanning of the position of the laser focus. The two adjacent hexapole elements are switched to a voltage configuration that accelerates the ions upwards to a drift tube, for time-of-flight mass-spectrometry. The ions are detected on a Multi-Channel-Plate (MCP) detector. The two detection zones in the synchrotron are marked by a laser beam in Fig. 3. During the collision measurements, the ammonia molecules are detected in detection zone II.

**Fig. 4:**
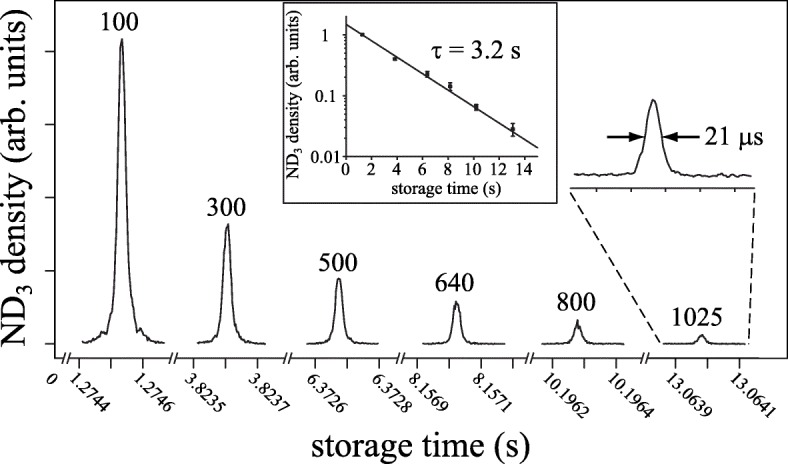
Time-of-flight measurements of ammonia molecules traversing the synchrotron. The numbers above the peaks denote the number of round-trips the molecules have completed at the time of measurement. The inset shows that the 1/*e* trapping time is 3.2 s. Reprinted with permission from Ref. [35] Ⓒ2010 APS

Figure 4 shows the ammonia signal measured in the synchrotron as a function of time. It demonstrates that a packet of ammonia molecules is still clearly visible even after completing more than 1000 round-trips, corresponding to a trapping time of over 13 s. In this time the molecules have traversed a distance of over a mile [35, 38, 39].

When the system is kept under vacuum for many weeks, the pressure reaches 5 ×10^−9^ mbar. Under these conditions the 1/e-lifetime of the stored packets is 3.2 s, determined equally by collisions with the background gas and black-body-radiation-induced transitions to non-trappable states [35]. In the experiments described in “Results” section the pressure is typically 2 ×10^−8^ mbar, resulting in a lifetime of 1.0 s.

**Fig. 5:**
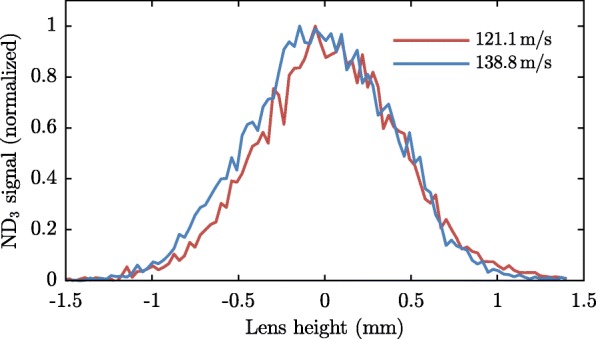
Laser height scans of ammonia packets traveling at 121.1 and 138.8 m/s at detection zone II

Figure 5 shows laser height scans of ammonia packets with velocities of 121.1 and 138.8 m/s, both after 90 round-trips. From these measurements, in combination with trajectory simulations, it is found that the emittance of the stored ammonia – describing the position and velocity spread of the molecules with respect to the so-called synchronous molecule – is [1 mm ·5 m/s]^2^ ·[4 mm ·1 m/s]. More details on the synchrotron, the beamline, and the trajectory-simulations of molecules through the synchrotron can be found in Zieger et al. [35, 38, 39].

## Collision partner beamline

### Longitudinal distribution

A schematic overview of the argon beamline is shown in Fig. 3. A supersonic argon beam is formed by releasing a high pressure (∼4 bar) gas into vacuum by a pulsed solenoid valve (Even Lavie type E.L.-5-2005 RT, HRR [40]) running at two times the cyclotron frequency of the synchrotron (153–175 Hz). To help keep the pressure in the source chamber below 1×10^−4^ mbar, an additional turbo-molecular pump provides a pre-vacuum of < 5×10^−4^ mbar to the turbo pumps of the source and detection chambers. The 5 mm diameter skimmer between the source and detection chambers and the 1.5 mm diameter skimmer between the detection and the synchrotron chambers select only the coldest part of the argon beam and ensures that the pressure in the synchrotron chamber remains below 2.2×10^−8^ mbar during operation.

In order to tune the velocity of the argon beam, the temperature of the valve housing can be varied between −150 °C and +30 °C. The temperature of the valve is regulated as follows: A flow of nitrogen gas is cooled down by passing it through a spiral tube immersed in liquid nitrogen, then passes a heater, and finally flows through a heatsink mounted onto the valve. The current flow through the heater is controlled by a Eurotherm 2408 PID-controller, which uses a thermocouple attached to the heatsink to read out its temperature. The time it takes for the valve temperature to reach its target temperature and stabilize can be between 45 min for a set temperature of 30 °C and 2 h for −150 °C. To avoid losing time, we typically operate the valve at a constant temperature throughout the day.

**Fig. 6:**
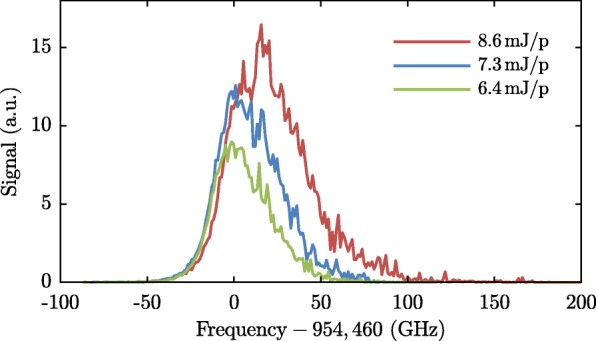
3+1 REMPI spectra at frequencies around the 3*s*^2^3*p*^6^(^1^*S*) to 3*s*^2^3*p*^5^(^2^*P*_1/2_)4*s*-transition in argon

The argon atoms can be detected at two locations: in detection zone I, approximately half-way the argon beamline, and in detection zone II, inside the synchrotron, where also the ammonia molecules are detected. UV laser pulses (5 ns long pulses with typically an energy of 10 mJ/pulse at *λ*=314 nm) are focused into the detection zones to ionize the argon atoms by 3+1 REMPI *via* the $3s^{2}3p^{5}\left ({~}^{2}P_{1/2}\right)4s\phantom {\dot {i}\!}$-state [41]. In detection zone I, the resulting ions are extracted upwards by a stack of electrostatic ion lenses and mass-selectively detected on an MCP detector. Inside the synchrotron (detection zone II) the ions are extracted by switching the two adjacent hexapole elements to the appropriate voltages, as discussed in the preceding section. Figure 6 shows spectra measured at three different laser powers illustrating that the transition is significantly broadened and shifted by the intensity of the laserpulses.

**Fig. 7:**
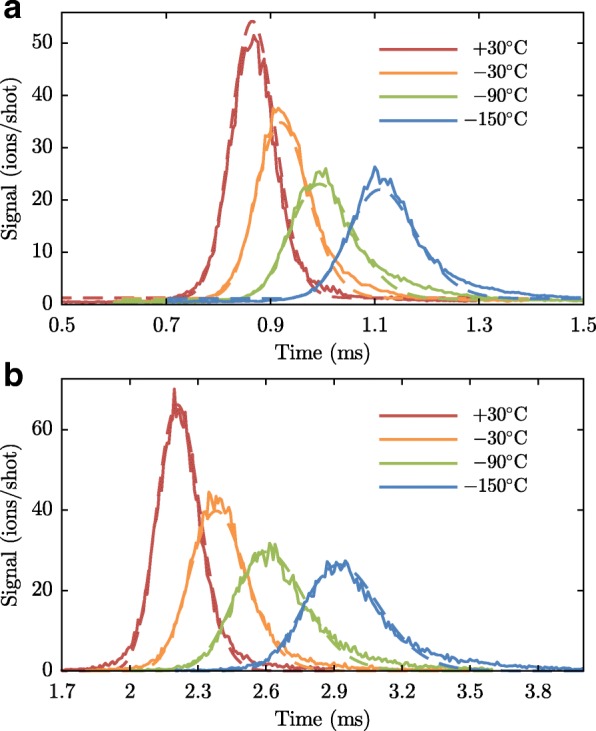
Argon time-of-flights as measured in detection zones I (**a**) and II (**b**), at different temperatures of the pulsed valve. For each temperature, the two time-of-flight measurements are fitted simultaneously using the model discussed in the text. The parameters resulting from the fits are listed in Table 1

In our experiment, described in the next sections, we operate the valve that releases the argon beam at four different temperatures (-150, -90, -30 and 30 °C) and use two ammonia velocities (121.1 and 138.8 m/s), which imply that the valve will operate at two repetition frequencies (153 and 175 Hz). To characterize the beams under these conditions, we have recorded many TOF profiles at both detection zones (I and II). The duration of the current pulse applied to the valve is limited to prevent multiple pulses due to bouncing of the plunger [40] (at high temperature) or a too high pressure in the source chamber (at low temperature). Typical measurements taken with the valve running at 153 Hz are shown in Fig. 7. Note that the MCP used in the first detection zone is rather old and its quantum efficiency has degraded over time. Whereas the density of the beam at the first detector is at least a factor of 10 higher than the density at the second detector, the count rate at both detectors is similar.

**Table 1:** Overview of the properties of the argon beam at different temperatures of the valve housing T _valve_, repetition rates *f*_rep_ (which are determined by the velocity of the stored ammonia molecules), ammonia velocities $v_{\mathrm {ND_{3}}}$, and durations of the current pulse applied to the valve *Δt*_pulse_. The second block lists properties of the beams measured at detection zone II: the relative beam intensity, mean arrival time, 〈*τ*〉, the width of the arrival time distribution *Δτ*, and the full width at half maximum (FWHM) length of the argon packet $L=\left \langle v_{Ar} \right \rangle \times 2 \sqrt {2\ln 2}~\Delta \tau $. Finally, the last block lists the parameters found from a simultaneous fit to the time-of-flight distributions measured at both detectors: the mean 〈*v*_*Ar*_〉 and standard deviation $\sigma _{v_{Ar}}$ of the velocity distribution, duration of valve opening time, and the effective distance between the valve and detection zone II

T _valve_ (°C)	*f*_rep_ (Hz)	$v_{\mathrm {ND_{3}}}$ (m/s)	*Δt*_pulse_ (*μ*s)	rel. int.	〈*τ*〉 (ms)	*Δτ* (*μ*s)	L (mm)	〈*v*_Ar_〉 (m/s)	$\sigma _{v_{\text {Ar}}}$ (m/s)	pw (*μ*s)	*Δz* (m)
+30	153	121.1	27.3	3.00 ±0.20	2.207	96	137	570.56	24.01	22.41	1.265
	175	138.8		2.74 ±0.18	2.205	94	134	571.41	24.09	22.48	1.265
−30	153	121.1	25.2	2.02 ±0.12	2.380	124	163	524.08	27.57	11.24	1.255
	175	138.8		1.91 ±0.11	2.378	122	160	524.73	27.30	13.73	1.254
−90	153	121.1	23.6	1.31 ±0.11	2.604	162	192	474.51	28.48	10.00	1.245
	175	138.8		1.21 ±0.10	2.597	159	189	475.40	29.22	10.00	1.245
−150	153	121.1	22.8	1.11 ±0.10	2.921	173	183	421.92	24.32	10.00	1.241
	175	138.8		1.00 ±0.09	2.916	175	185	423.40	24.98	10.00	1.242

The longitudinal properties of the argon beam are modelled as follows. In general, the spatial density distribution *n*_Ar_(*z*|*v*,*t*) of argon atoms with a velocity between *v* and *v*+d*v* at a time *t* can be described by

3 $$ n_{\text{Ar}}(z|v,t) = N ~ f^{v}(v) ~ \mathrm{d}v ~ f^{z}(z|v,t),  $$

where *N* is the number of atoms in the packet, *f*^*v*^(*v*) is the time-independent, normalized, velocity distribution, and *f*^*z*^(*z*|*v*,*t*) is the normalized position distribution of argon atoms with a velocity between *v* and *v*+d*v* at time *t*. We assume the velocity distribution *f*^*v*^(*v*) to be given by the normal distribution *g*^*v*^(*v*|〈*v*〉,*σ*_v_) with average 〈*v*〉 and standard deviation *σ*_v_. The position distribution *f*^*z*^(*z*|*v*,*t*) at a certain time *t* can be extrapolated from the position distribution at the source *f*^*z*^(*z*|*v*,*t*=0). This initial distribution is assumed to be a normal distribution with 〈*z*〉=*z*_valve_. From the opening time of the valve and the velocity of the atoms in question, the standard deviation is given by *v* times *pw*. Here *pw* stands for pulse width, i.e. the time that the valve is open. In time, the center of the initial position distribution moves with velocity *v* while the shape remains constant, as all the atoms have the same velocity *v*. Thus, the position distribution at a certain time *t* is given by the normal distribution *g*^*z*^(*z*|*z*_valve_+*vt*,*v* *pw*). The measured signal *S*(*z*,*t*) at position *z* and at time *t* is proportional to the argon density, which is found by integrating *n*_Ar_(*z*|*v*,*t*) over all velocities:

4 $$  S(z,t) \propto \int\limits_{-\infty}^{+\infty} g^{v}(v|\left\langle v \right\rangle, \sigma_{\mathrm{v}}) ~ g^{z}(z|z_{\text{valve}}+vt, v~pw) ~ \mathrm{d}v.  $$

Equation 4 is simultaneously fitted to the TOF profiles measured at detection zone I and detection zone II. The fit parameters, 〈*v*〉, *σ*_v_, *pw*, and *z*_valve_, are determined by minimizing the weighted sum of the squares of the residuals, and are presented in Table 1.

**Fig. 8:**
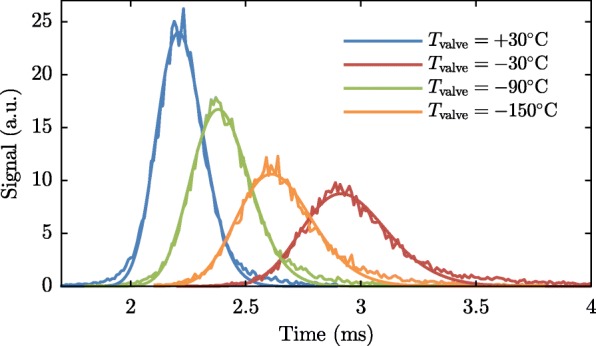
Argon time-of-flight measurements (with fits) in detection zone II at the different valve temperatures for the purpose of argon beam intensity calibration

In order to infer the collision cross sections with high precision, it is crucial to determine the argon beam intensities accurately. Therefore, time-of-flight measurements were made at detection zone II (close to where the collisions will happen), at each of the four valve temperatures and two repetition rates, in a single day. Care was taken to keep the laser power at 8.00±0.05 mJ/p. These measurements are shown in Fig. 8. They are fitted with the arrival time distribution for a beam with a normal velocity distribution originating from a point source, given by

5 $$ S(t)=S_{0}~\exp\left\lbrace -\left(\frac{1}{t}-\frac{1}{\left\langle\tau\right\rangle}\right)^{2}/\left(2\frac{\Delta\tau^{2}}{\left\langle\tau\right\rangle^{4}}\right)\right\rbrace,   $$

where *S*_0_ is the peak signal, 〈*τ*〉 the mean arrival time, and *Δτ* a measure of the width of the distribution similar to a standard deviation. The distribution resembles a normal distribution that is unevenly stretched along the time axis: the slower part of the beam needs a longer time to arrive at the detection zone, and therefore has more time to disperse. The relative beam intensities are given by the fit maxima (S_0_), normalized to the argon beam at *T*_valve_=−150 °C. The resulting calibrations are also presented in Table 1.

### Transverse distribution

The geometry of the beamline, i.e., the skimmers and the gap between the hexapole rods through which the argon beam enters the synchrotron, determines partially, but not completely the transverse spatial and velocity distributions of the argon beam. Following the recommendations given in Ref. [40], we have installed a skimmer with a large aperture (Beams dynamics, type II, 5 mm) at a distance of 200 mm in front of the valve. The effective size of the source, i.e., the transverse size of the argon beam when the density has dropped to such extent that collisions cease to be important (the freezing point), is smaller than the aperture of the first skimmer. Hence, this effective size, together with the second skimmer and the gap between the electrodes, determines the transverse spatial and velocity distribution of the argon beam in the synchrotron.

**Fig. 9:**
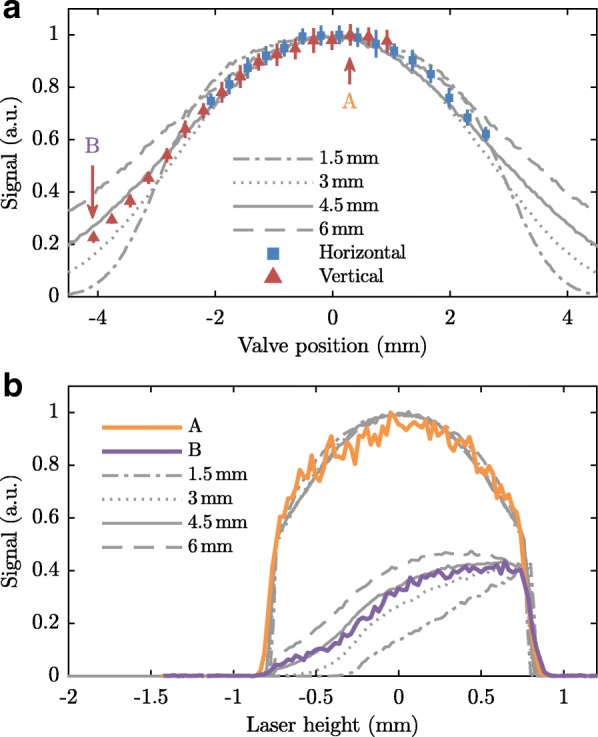
**a** Argon signal in detection zone II as function of the horizontal (blue squares) or vertical (red triangles) position of the valve. Error bars represent standard errors of the means. **b** Scan of the vertical position of the laser focus when the valve is positioned close to the center (corresponding to arrow “A” in panel **a**) or 4 mm below the center (corresponding to arrow “B” in panel **a**). The grey lines in (**a**) and (**b**) show simulations with different values of the effective size of the beam source

To determine the effective size of the source, we have performed two types of measurements: (1) we have recorded the argon signal inside the synchrotron while scanning the horizontal and vertical positions of the valve, and (2) we have scanned the height of the laser focus for two positions of the valve. The results of both measurements are shown in Fig. 9. The blue squares in Fig. 9a show the signal when the valve is displaced in the horizontal direction, while the red triangles show the signal when the valve is displaced in the vertical direction. Note that, since the skimmer is located at ∼80% along the path between the valve and the detection zone, a displacement of 4 mm of the valve results in a beam displacement of ∼1 mm in the detection zone. At two vertical positions of the valve, the vertical distribution is measured by scanning the laser focus as shown in Fig. 9b. The orange curve is measured while the valve is close to the center position (indicated by the arrow “A” in Fig. 9a), while the purple curve is measured when the valve is 4 mm down from the center (indicated by the arrow “B” in Fig. 9a).

These measurements are compared with simulations that calculate the trajectories of argon atoms through the machine. The simulation starts by initializing argon atoms with random positions and velocities, given by Gaussian distributions. The atoms then fly in a straight line from the valve to the detection zone. They are counted if they fly through both skimmers and arrive at the laser focus. The results of simulations performed with effective source sizes of 1.5, 3, 4.5, and 6 mm are depicted by the gray lines in Fig. 9. The widths of the velocity distributions are unimportant, as the divergence of the beam at the source is much larger than what is accepted by the skimmers, and are set to be >10 m/s. From the measurements, we conclude that the effective size of the source is ∼4.5 mm.

**Fig. 10:**
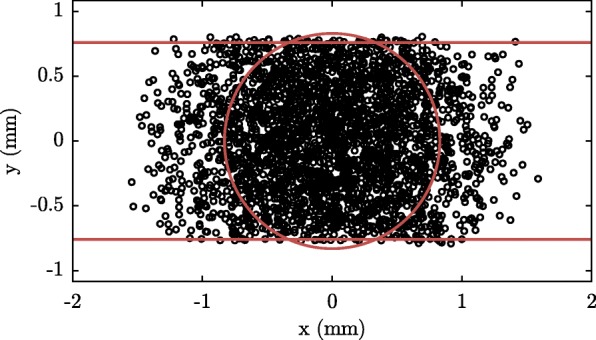
Transverse position distribution of simulated argon atoms at the longitudinal position where the atoms first collide with the stored ammonia molecules. The horizontal lines reflect the cut-off of the electrodes that make up the hexapole element through which the argon beam first enters the synchrotron. The circle approximates the circular cut-off due to the second skimmer. The resulting flattened circle shows the transverse shape of the argon beam that is used in trajectory simulations of ammonia molecules

Figure 10 shows a simulation of the transverse distribution of the argon beam at the longitudinal position where the atoms (first) collide with the stored ammonia molecules. This simulation uses the parameters found from the experiment. In order to simplify the analysis of our collision experiment, we will approximate the beam by a cylinder with a diameter of 1.66 mm, indicated by the red circle in Fig. 10; the density of the gas within this cylinder is assumed to be independent of the radial direction and is taken to be equal to the peak density. In this way, the number of atoms in the model equals the number of atoms in the experiment. To account for the effect of the gap, the distribution is limited to 1.52 mm in the vertical direction. The size of the beam increases by 5% over the course of 40 mm. The intensity decreases concomitantly so that the number of molecules remains constant.

In the vertical plane, the overlap of the argon beam with respect to the synchrotron is optimized by using the height scans discussed above. In the horizontal plane, the optimal alignment is more difficult to find. In the collision experiments presented in the next sections, we deliberately aligned the argon beam inwards with respect to the equilibrium orbit of the stored ammonia molecules, such that it intersects the path of the stored molecules twice: once close to the detection zone in the synchrotron, and once further downstream. As a result of this alignment a measurement of the loss rate as a function of delay between the trigger of the valve that releases the argon beam and the arrival time of the ammonia molecules in the detection zone displays two maxima rather than one. From the delay between two maxima we can infer the displacement.

## Results

### Collisions at a specific collision energy

We now arrive at the heart of this paper: the collision measurements. As explained in “Molecular synchrotron” section, we store multiple packets of ammonia molecules in a synchrotron while sending in beams of argon that are made to collide with some of the packets–the probe packets. Packets that do not encounter the co-propagating beam–the reference packets–provide a simultaneous measurement of background loss. In the experiments discussed here, the valve that releases the argon beams runs at twice the cyclotron frequency such that every tenth ammonia packet encounters a fresh argon beam every round trip.

**Fig. 11:**
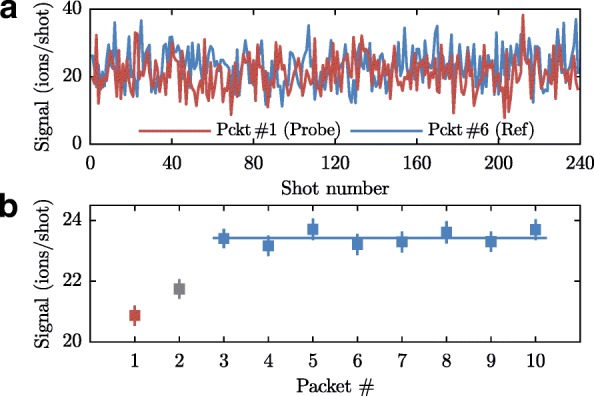
**a** Single-shot measurements of ammonia packets after 90 round-trips. The red trace corresponds to the probe packet, the blue trace to one of the reference packets. **b** 240-shot averages of the probe packet (red), a discarded reference packet (grey), and 8 reference packets (blue). The error bars denote standard errors of the means. The blue line depicts the average of the 8 reference packets

Figure 11 shows a typical measurement of an experiment where ammonia molecules, revolving in the synchrotron with a mean velocity of 121.1 m/s, are detected after 90 (and a quarter) round-trips. These molecules are made to collide with beams of argon atoms with mean velocities of 474.5 m/s. Typically, we take data in blocks of 4 min corresponding to 2400 individual measurements, taken at a rate of 10 Hz. Measurements that are taken at the same delay with respect to the partner beam are grouped together, resulting in ten sets of 240 measurements each. The measurements in each set are averaged and further analysed. The top panel of Fig. 11 shows two traces corresponding to the probe packet (red) and one of the reference packets (blue). The noise on the measured traces has a standard deviation of 5.3 ions per shot which is only slightly larger than the $\sqrt {23.4}=4.8$ ions per shot expected for a Poissonian distribution. The difference is attributed to noise added by the MCP detector. The lower panel of Fig. 11 shows the averages for each of the 10 sets, along with the standard errors of their means ($\text {SE}=\sigma /\sqrt {n}$). The signal of the probe packet (#1) is about 11% smaller than the average of packets #3–10, corresponding to a loss rate of k _col_=(1.28±0.18)×10^−3^ per round-trip. As observed, the argon beam also has some overlap with packet #2 in this particular experiment; this packet is therefore discarded. Depending on the timing of the partner beam, which determines whether the trapped ammonia molecules encounter the rising, middle or trailing part of the argon beam, packets #2, #3, #9, and/or #10 are discarded.

### Model to extract the relative cross section from the measured loss rates

The measurements presented in the previous paragraph demonstrate the unique properties of a molecular synchrotron for studying collisions, in particular the high sensitivity owing to the fact that collision signal can be accumulated over the long time that the molecules are stored. Our next goal is to determine the relative (integral, total) cross sections from the measured loss rates. In order to determine these, a detailed understanding of how the ammonia molecules move through the argon packets is required. Which parts of the argon packet do the ammonia molecules encounter? What are the velocities of the argon atoms in these parts? What is the density of the argon beam in these parts? To answer these questions, trajectory simulations will be performed. The goal of the simulations is to find (1) an expectation value for the amount of encountered argon atoms, and (2) the corresponding distribution of collision energy.

The number of argon atoms encountered can be found by integrating the argon density over the volume probed by the ammonia molecules as they revolve around the synchrotron. As a warming up exercise, we will first consider the simple case of an ammonia molecule flying along a path C through a homogeneous gas of argon atoms, with 〈*v*_Ar_〉=0 and number density *n*. Throughout this section, the position and velocity of the ammonia molecule will be denoted by $\vec {r} = (x, y, z)$ and $\vec {v} = (v_{x}, v_{y}, v_{z})$, respectively. We will first assume that the collision cross section *σ*_tot_ is independent of relative velocity. In this case, the average number of argon atoms encountered by the ammonia molecule is

6 $$ \left\langle N_{\text{coll}} \right\rangle = \int\limits_{C} \sigma_{tot} \, n \, \mathrm{d}s = \int\limits_{t_{i}}^{t_{f}} \sigma_{\text{tot}} \, n \, |v(t)| \, \mathrm{d}t = L \, \sigma_{\text{tot}} \, n,  $$

where the path *C* is parametrized by

7 $$ C: z(t), v(t), \quad t_{i}\leq t \leq t_{f},  $$

and where

8 $$ L=\int\limits_{t_{i}}^{t_{f}} |v(t)| \, \mathrm{d}t  $$

is simply the path length.

In the actual experiment, of course, the argon gas is not homogeneously distributed. On the contrary: since the argon beam results from a supersonic atomic beam, it will have a distribution that depends strongly on position and time. Furthermore, the velocity distribution of the argon atoms is important: both the effective path length $\mathrm {d}l=|\vec {v}_{\text {rel}}(t)| \, \mathrm {d}t$ and the collision cross section *σ*_tot_(*E*_col_) depend on the velocity of the argon atoms. Thus, we need the argon atom number density $n(\vec {r}|\vec {v}_{\text {Ar}},t)$ as function of position and time for any argon velocity, so that we can find the number of argon atoms that are encountered by an ammonia molecules using

9 $$ \left\langle N_{\text{coll}} \right\rangle = \left\langle \sigma_{\text{tot}} \right\rangle \int\limits_{t_{i}}^{t_{f}} \int\limits_{-\infty}^{+\infty} n(\vec{r}|\vec{v}_{\text{Ar}},t) \, \mathrm{d}t \, \mathrm{d}v_{\text{rel}},  $$

where 〈*σ*_tot_〉 is the result of averaging the collision cross section *σ*_tot_(*v*_rel_) over the velocities of all argon atoms that are encountered. In our experiments, this average is the only thing we can ever measure. We will now make this general expression more specific in order to obtain an expression that reflects the conditions of our experiment.

As discussed earlier, the longitudinal distribution of the argon beam can be described by multiplying a gaussian velocity distribution with a gaussian spatial distribution, as expressed in Eq. 4. Transversely, the argon beam is assumed to be homogeneously distributed over the area that makes up the transverse cross section of the argon beam. As the beam is divergent, the size of this area depends on the longitudinal position and will be denoted by *S*(*z*). Note that Fig. 9b reveals that the profiles are actually not entirely flat, but the approximation is sufficiently accurate for our purpose. Since contributions of the transverse velocity components of the argon atoms to the collision energy are negligible, we assume *v*_Ar,x_=*v*_Ar,y_=0 so that $v_{\text {Ar}} \equiv \left | \vec {v}_{\text {Ar}} \right | v_{\mathrm {Ar,z}}$. A general expression of the argon beam is then given by

10 $$ n_{\text{Ar}}(\vec{r}|v_{\text{Ar}},t) = N \, f^{v}_{\text{Ar}}(v) \, \mathrm{d}v \, f^{z}_{\text{Ar}}(z|v,t) / S(z),  $$

for (*x*,*y*)∈*S*(*z*), where *N* is the number of atoms in the packet.

The transverse cross section *S*(*z*) can be parameterized by

11 $$ S(z) = S(z_{0}) \cdot (1+(d(z-z_{0}))^{2}),  $$

where *S*(*z*_0_) is the cross section at the position where the argon beam crosses the equilibrium orbit (the first time), and where *d* describes the divergence of the beam. The cross section of the beam at *z*_0_ is shown in Fig. 10. Both *S*(*z*_0_) and *d* are determined from the trajectory calculations described earlier.

As the total number of atoms in the beam is not of interest, we will write the argon beam density as:

12 $$ \begin{aligned} n_{\text{Ar}}(\vec{r}|v_{\text{Ar}},t) & \equiv n_{0} \, L \cdot f^{v}_{\text{Ar}}(v_{\text{Ar}}) \, \mathrm{d}v_{\text{Ar}} \cdot f^{z}_{\text{Ar}}(z|v_{\text{Ar}},t) \cdot q(\vec{r}), \end{aligned}  $$

where *n*_0_ is the peak number density in the packet at *z*_0_ and a *L* is a measure for the length of the (Gaussian-shaped) packet, respectively, such that *n*_0_ *L*=*N*/*S*(*z*_0_) is the column density of the argon packet at *z*_0_. The newly introduced function

$$q(\vec{r}) = \left\{ \begin{array}{ll} \frac{1}{1+(d(z-z_{0}))^{2}} & \text{if} \enspace (x,y) \in S(z) \\[0.5ex] 0 & \text{otherwise}, \\ \end{array} \right. $$

is a shorthand to reduce the length of future equations. This function describes whether the transverse position of the ammonia molecule is located within the argon beam (or not), and also accounts for the decreasing density of the argon beam due to its divergence.

Finally, since we are interested in the number of argon atoms encountered as a function of the collision energy rather than as a function of argon atom velocity, we perform a change of integration variable from *v*_Ar_ to *v*_rel_ using the relation

13 $$ v_{\text{rel}} = | v_{x} \hat{x} + v_{y} \hat{y} + (v_{z}-v_{\text{Ar}}) \hat{z} |.  $$

Putting all of this together, we find for the expectation value for the number of encountered argon atoms

14 $$  \begin{aligned} \left\langle N_{\text{coll}} \right\rangle (t_{\text{valve}}) & = \left\langle \sigma_{\text{tot}} \right\rangle n_{0} \, L \, \int\limits_{t_{i}}^{t_{f}} \mathrm{d}t \int\limits_{-\infty}^{+\infty} \mathrm{d}v_{\text{rel}} \dots \\ & \left\lbrace f^{z}_{\text{Ar}}(z|v_{\text{Ar}},t) \cdot f^{v}_{\text{Ar}}(v_{\text{Ar}}) \cdot v_{\text{rel}} \cdot \frac{\mathrm{d}v_{\text{Ar}}}{\mathrm{d}v_{\text{rel}}} \cdot q(\vec{r}(t)) \right\rbrace \\ & \equiv \left\langle \sigma_{\text{tot}} \right\rangle n_{0} \, L \, \int\limits_{-\infty}^{+\infty} g^{N_{\text{coll}}}(v_{\text{rel}}) \, \mathrm{d}v_{\text{rel}} \\ & \equiv \left\langle \sigma_{\text{tot}} \right\rangle n_{0} \, \beta \, L \int\limits_{-\infty}^{+\infty} f^{N_{\text{coll}}}(v_{\text{rel}}) \, \mathrm{d}v_{\text{rel}}\\ & = \left\langle \sigma_{\text{tot}} \right\rangle n_{0} \, \beta \, L, \end{aligned}  $$

where a few new quantities have been introduced. Firstly, $\phantom {\dot {i}\!}g^{N_{\text {coll}}}(v_{\text {rel}}) \, \mathrm {d}v_{\text {rel}}$ is the result of the path integral over the argon density distribution. In order to aid the interpretation of this result, however, it is separated out into a parameter *β* and distribution $\phantom {\dot {i}\!}f^{N_{\text {coll}}}(v_{rel}) \, \mathrm {d}v_{rel}$. The former, called the beam-overlap, is a parameter between 0 and 1 that describes the effective length of the part of the argon packet that is probed by the ammonia molecules, relative to the total length of the packet. The latter is a normalized distribution function that describes the distribution of the relative velocities of the argon atoms that are encountered. This function determines the energy resolution of the measured cross section.

The model is implemented as follows. Firstly, the trajectory simulations calculate the beam-overlap, in the form of *g*^*N*^(*v*_rel_) d*v*_rel_. The cross section and beam densities are not included. The beam-overlap is calculated by numerically integrating the path of the synchronous molecule from *t*_*i*_ to *t*_*f*_. The simulation calculates, on every (variably-sized) time-step *Δt*_*i*_, for every velocity *v*_rel,j_ on a grid with spacing *Δv*_rel_:

15 $$ \begin{aligned} & g^{z}_{\text{Ar}} \left\lbrace z(t)|z_{\text{valve}}+v_{\text{Ar}}(v_{\text{rel}},\vec{v}(t)) \cdot (t-t_{\text{valve}}), v~pw \right\rbrace \cdot \dots \\ & g^{v}_{\text{Ar}} \left\lbrace v_{\text{Ar}}(v_{\text{rel}},\vec{v}(t)) | \left\langle v_{\text{Ar}} \right\rangle, \sigma_{\mathrm{v_{Ar}}} \right\rbrace \cdot \dots \\ & \frac{\mathrm{d}v_{\text{Ar}}}{\mathrm{d}v_{rel}} \, q \left\lbrace \vec{r}(t) \right\rbrace \, v_{\mathrm{rel,j}} \, \Delta t_{i} \, \Delta v_{rel}, \end{aligned}  $$

where $g^{z}_{\text {Ar}}$ and $g^{v}_{\text {Ar}}$ are the normal distributions from Eq. 4, and adds it to a histogram over *v*_rel_. This provides us with the relative velocity distribution of the beam-overlap, $\phantom {\dot {i}\!}g^{N_{\text {coll}}}(v_{\text {rel}})$, which is then integrated over *v*_rel_ to find the beam-overlap, *β*. The simulations were performed using ammonia distributions with different temperatures and by using either a complete model that derives the force on the ammonia molecules from the electric field in the synchrotron, which was calculated by SIMION [42], or a toy model that assumes a linear restoring force towards the synchronous molecule, using the trapping frequencies from Zieger et al. [39]. As the results of these simulations did not differ significantly from each other, we have decided to perform all further calculations using the toy model and assuming a zero temperature for the ammonia molecules. Since in this case each round-trip is identical, only a single round-trip is simulated. Note that 〈*N*_coll_〉 is then simply the number of collisions per round-trip, *k*_col_.

### Measuring the collision cross section a function of collision energy

We now have all necessary tools to reach our final goal; measuring the relative, total, integrated collision cross sections as a function of energy. The collision energy can be tuned in three different ways: by varying the velocity of the stored ammonia packets, by varying the temperature of the pulsed valve that releases the argon atoms, and by varying the timing of the supersonic argon beam with respect to the stored ammonia packets. Unfortunately, when we vary the collision energy, other parameters that influence the loss rates of the stored molecules will change as well. For instance, by changing the temperature of the valve that releases the argon beam, the average velocity of the beam will change, but so will the intensity, the velocity distribution and the beam overlap. When we change the velocity of the stored ammonia molecules, the argon beam will remain the same but the beam overlap will change. Luckily, the model that was derived in the previous section tells us how to take all these effects into account.

We will first look at collision measurements as a function of the delay between the trigger of the valve that releases the argon atoms and the arrival time of the ND_3_ probe packet in the detection zone. This delay determines whether the ammonia molecules collide with atoms located more in the leading or trailing end of the argon packet, or, in fact, whether they collide at all. As the flight time from the valve to the synchrotron is much larger than the opening time of the valve, there is a strong correlation between the position of the argon atoms and their velocity. Hence, the delay determines the velocity of the argon atoms that are encountered by the ammonia beam. Note that in our experiments, the relative velocities are such that ammonia molecules only see part (20–30%) of the argon packet during the time they spend in the collision zone.

**Fig. 12:**
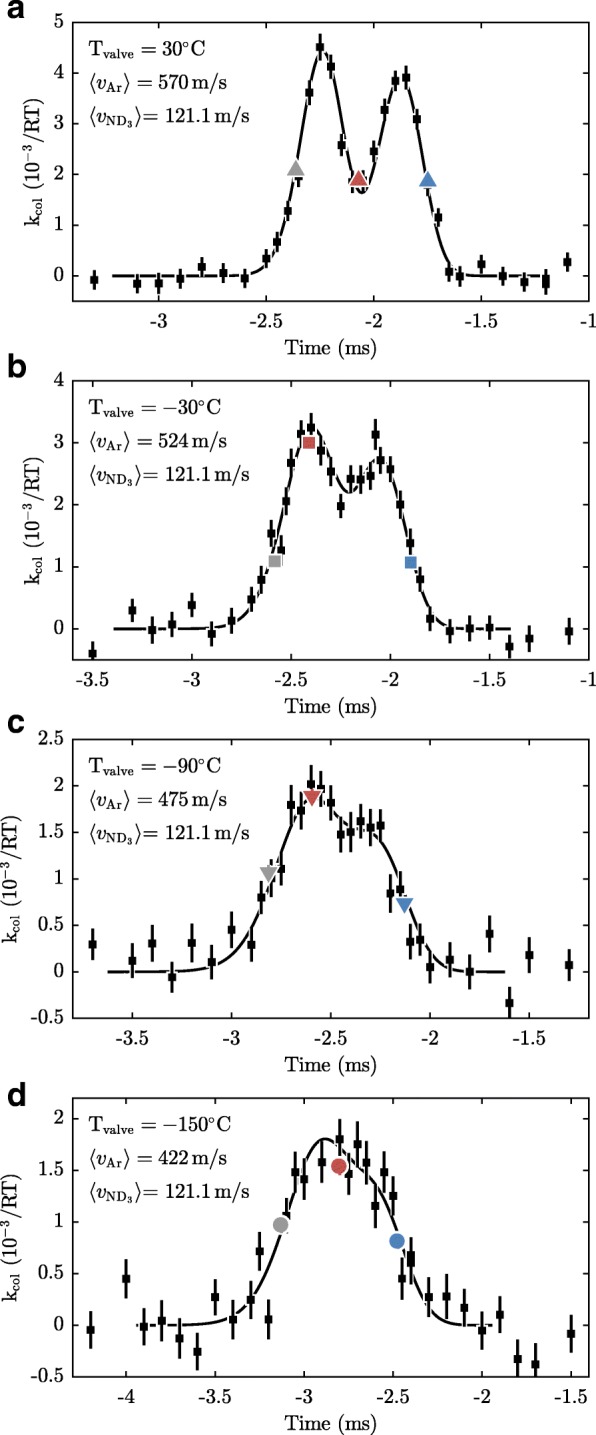
Measured loss rate as a function of the delay between the trigger of the valve and the arrival time of the ammonia packets at the detection zone, for v$\protect \phantom {\dot {i}\!}{~}_{\text {ND}_{3}}=121.1$ m/s. Panels **a**–**d** depict measurements with different temperatures of the valve housing that result in different average velocities as indicated in the top left corners. The black squares represent measurements containing 2400 shots each, while the coloured points represent measurements containing 21,600 shots each. The error bars, for the coloured points obscured by the symbols, denote the standard errors. The black lines show the results of simulations described in “Model to extract the relative cross section from the measured loss rates” section, scaled to fit the data in each panel. The reduced *χ*^2^’s of the fits are 0.93, 1.01, 1.09, and 1.31 for panels **a**–**d**, respectively

**Fig. 13:**
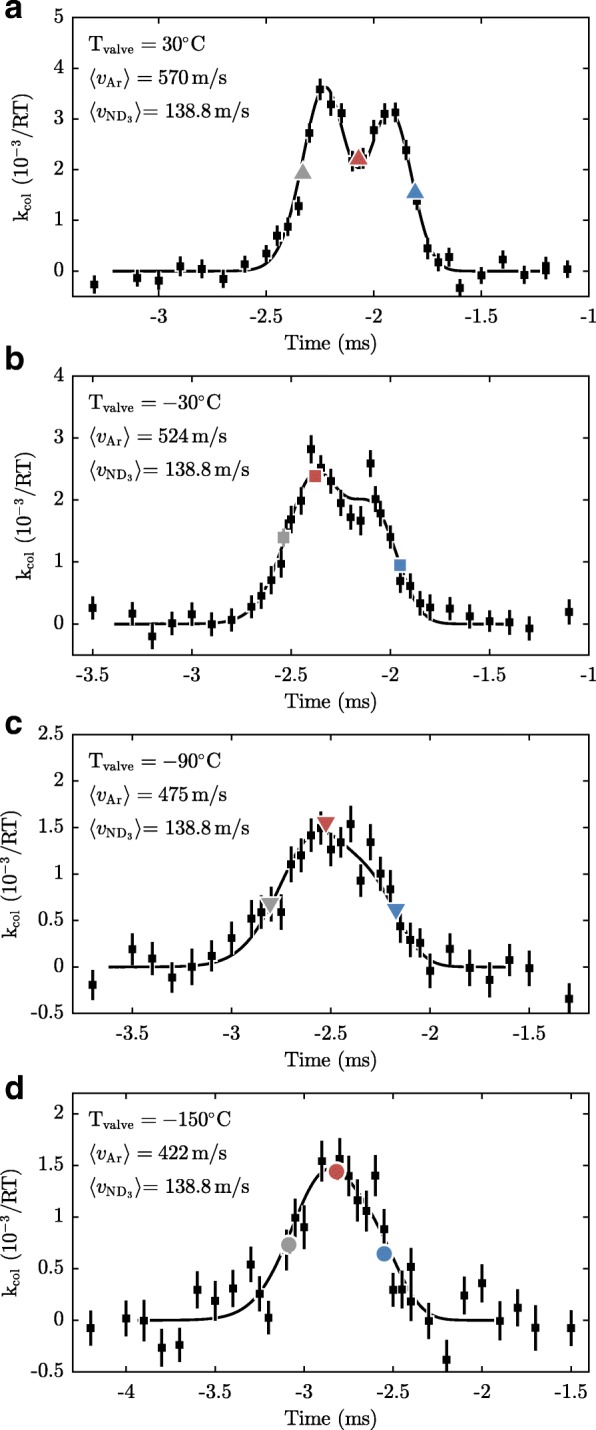
Same as Fig. 12, but for v$\protect \phantom {\dot {i}\!}{~}_{\text {ND}_{3}}=138.8$ m/s. The reduced *χ*^2^’s of the fits are 0.98, 0.89, 0.79, and 1.39 for panels **a**–**d**, respectively

Figures 12 and 13 show the ammonia loss rate measured as a function of the delay, for ammonia with velocities of 121.1 m/s and 138.8 m/s, respectively. The temperature of the valve that releases the argon atoms is kept at temperatures between −120 to 30°C as indicated in the figures. Each data point is the result of a 4 min collision measurement, such as the one depicted in Fig. 11. To be robust against possible drifts of the argon density, the data were taken while toggling between the two ammonia speeds after every data point and picking the timings from a list in a random order. As observed, the scans feature two peaks rather than one, resulting from the fact that the argon beam intersects the synchrotron at two distinct locations. These peaks become less well resolved as the argon packets become slower and concomitantly longer.

Additional data were taken at three specific timings of the argon beams, shown in Figs. 12 and 13 as the blue, red and grey symbols. The blue data points are measured using a short time delay between the valve and the arrival time of ammonia packet. Hence, in this case, the ammonia molecules probe the fast part of the argon beam. The red points are measurements that probe the central part of the argon beam, while the grey points are measurements that probe the slow part. Each of these measurements is the result of 21,600 shots, corresponding to a measurement time of 36 min per point. To detect and correct for possible drifts of the Ar beam density during the measurements, we cycled nine times through the six different configurations. No significant drifts were detected.

The solid curves in Figs. 12 and 13 show results of simulations of our experiment using the model discussed in the previous section. The simulation uses as input the velocity and equilibrium radius of the synchronous molecule taken from simulations of the synchrotron [39], and the longitudinal and transverse position and velocity distributions of the argon beams. The horizontal displacement of the argon beam relative to the equilibrium orbit of the ammonia molecules was varied in order to optimize the fit (simultaneously for all 8 curves). From this we find (1) that the argon beam is displaced by 1.6 mm from the ammonia molecules’ equilibrium orbit, which corresponds to a 58 mm distance between the two points around which the particles interact, and (2) the position of the crossing point with respect to detection zone II. The former determines the time difference between the peaks in Figs. 12 and 13, while the latter determines an off-set for the time axis. Furthermore, the simulations are scaled vertically to match the experimental data for each of the 8 curves individually. As seen from the figure, the simulations describe the measurements very well. For instance, the width of the distributions is well reproduced and so is the difference in signal at the two peaks. This agreement confirms that we have an excellent understanding of the experiment.

**Fig. 14:**
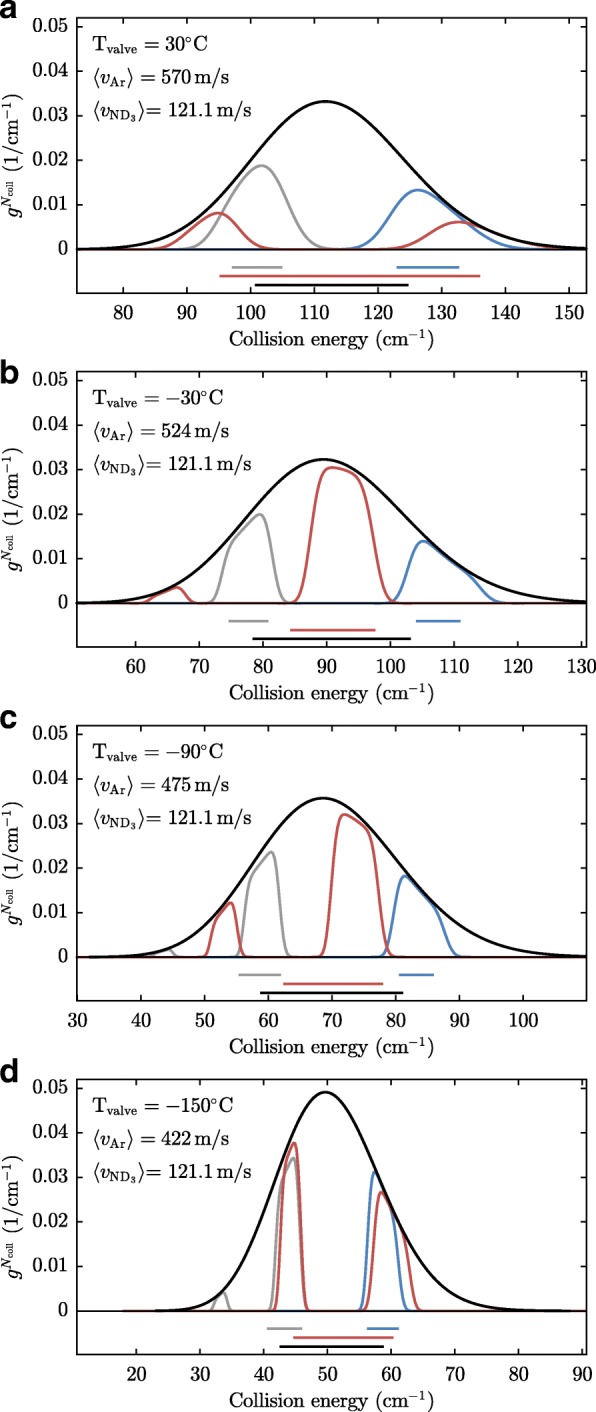
**a**-**d** Collision energy distributions determined from simulations for collision experiments with $v_{ND_3}=121.1$ m/s and *v*_*Ar*_ as indicated. The black curves represent the distribution for collision measurements that combine the measurements at each valve timing, i.e. all the black squares in Figs. 12 and 13. Since in this way the entire argon packet is probed, the distribution corresponds simply to the longitudinal velocity distribution of the argon beam at a particular temperature. The grey, red, and blue curves represent the collision energy distributions for the collision measurements indicated by the grey, red, and blue data points in Figs. 12 and 13. The grey, red, blue, and black bars in the bottom of each graph represent the widths of the distributions, as they are displayed by the horizontal error bars in Fig. 16

**Fig. 15:**
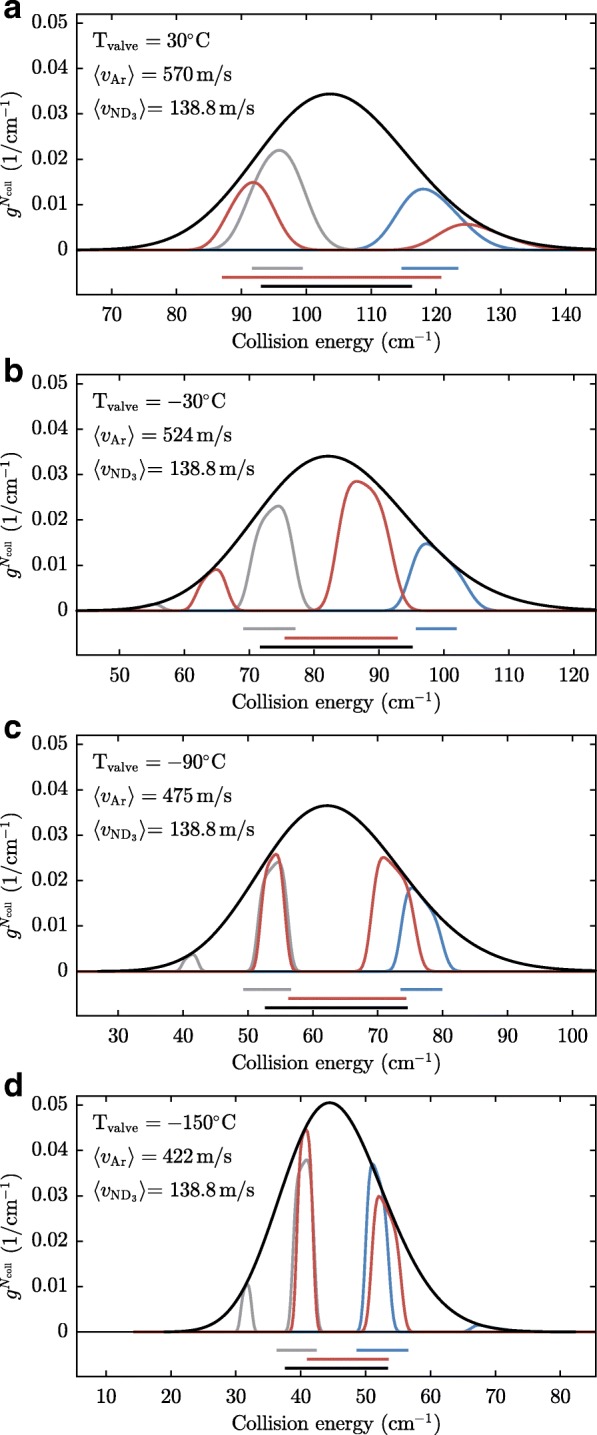
**a**-**d** Same as Fig 14**a**-**d**, but for $v_{ND_3}=138.8$ m/s

From the simulations we retrieve the distributions of the relative velocities of the encountered argon atoms, which are shown in Figs. 14 and 15. Each panel in Figs. 14 and 15 corresponds to a panel in Fig. 12 or Fig. 13. The black curves in Figs. 14 and 15 show the distribution of collision energies integrated over the entire argon beam – this distribution is relevant when the cross section is found by scaling the simulation to the entire delay scan shown in Figs. 12 and 13. The blue, red and grey curves in Figs. 12 and 13 show the collision energy distributions for the data points taken in the front, middle and back part of the argon gas pulse, respectively. As in this case only part of the argon beam is probed, the distributions are more sharply peaked. As expected, the blue curve is centered at higher collision energies than the average, while the grey curve is centered at lower collision energies. The red curve is bi-modal which is an obvious disadvantage of the chosen alignment of the argon beam.

**Fig. 16:**
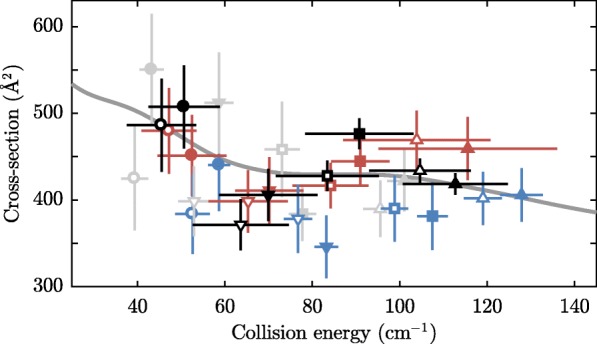
Total, integrated, ND_3_+Ar collision cross section versus collision energy. The meaning of the black, grey, red, and blue points is described in the main text. The vertical error bars denote standard errors, the horizontal error bars denote the collision energy distributions as depicted in Figs. 14 and 15. The grey line shows the result of scattering calculations[43], convoluted with a normal distribution with a standard deviation of 5 cm ^−1^. The measurements are collectively fit to this calculation with a single global scaling factor that represents the density of the argon beam at T=−150 °C, which is found to be 7.8 ×10^9^ cm ^−3^. $\chi _{\text {red},\nu =31}^{2}=1.3$. Reprinted with permission from Ref. [32] Ⓒ2018 APS

The bars below Figs. 14 and 15 depict the standard deviation of the collision energy distributions, which are a measure for the energy spread of the collisions which are probed in our measurement. Clearly, the interpretation of the standard deviation is not obvious in the case of bi-modal distributions, but we will use these for lack of a better measure.

We are now ready to retrieve the relative cross section as function of energy. Rewriting Eq. (14), we obtain an equation that relates the cross section to the measured loss rates:

16 $$ \left\langle \sigma_{\text{tot}} \right\rangle = \frac{n_{0,c} \, n_{\mathrm{0,rel}} \, \beta \, L}{k_{\text{col}}},  $$

where the argon beam densities (at position z_0_) are written as the product of *n*_0,*c*_, the absolute density of the argon beam at *T*_valve_=−150 °C and *f*_rep_=153 Hz, and *n*_0,rel_, the relative argon beam densities as presented in Table 1, together with the length of the argon pulses *L*. The beam overlap, *β*, is taken from the simulations.

Before presenting our final result, there is one more thing to consider. In our experiment we determine the loss rate of stored ammonia molecules due to collisions with argon atoms. Although most collisions lead to loss, a small (∼10%) but significant fraction of elastic collisions takes place at such large distances that the ammonia molecules remain trapped in the ring. To correct for this effect we multiply the found loss rates with an energy dependent correction factor. This correction factor is calculated by combining our knowledge of the trapping potential with the differential collision cross section (the cross section as a function of scattering angle) from quantum close-coupling calculations of ND_3_ + Ar collisions, performed by Loreau and van der Avoird [43]. A detailed explanation is given in the supplementary material to Van der Poel et al. [32].

Figure 16 presents the resulting cross sections together with their uncertainties and collision energy distributions as determined by the simulations. The blue, red, and gray points in Fig. 16 are obtained from measurements at the front, center, and back of the argon beams at four different temperatures of the valve. The black data points result from averaging over all timings; these are the scaling factors obtained by fitting the simulated delay scans to the measurements shown in Figs. 12 and 13. The open and closed symbols are measured with ammonia molecules that have a velocity of 138.8 and 121.1 m/s, respectively. The uncertainties in the cross sections, in the range of 7–14%, are a combination of the uncertainties of the relative argon intensities (presented in Table 1, typically between 6–9%) and the uncertainties in the measured loss rates (as shown in Figs. 12 and 13, typically between 2–11%). The fact that we find consistent results when the collision energy is changed in different ways, gives us great confidence in the measured cross sections. Note that the loss rates from which the cross sections are derived vary from 0.5 ×10^−3^ to 3 ×10^−3^ per round-trip. We attribute the small differences observed between the different data sets to the approximations made in the model – particularly, the fact that the argon beam is assumed to have a gaussian velocity distribution.

The solid line also shown in Fig. 16 is the result of theoretical calculations performed by Loreau and van de Avoird [43], convoluted with a normal distribution with a standard deviation of 5 cm ^−1^. The measurements are collectively fit to this calculation with a single global scaling factor that represents the density of the argon beam at T=−150 °C, which is found to be 7.8 ×10^9^ cm ^−3^ about 1.2 m down-stream from the valve. This density is in agreement with a crude estimate of the density from the REMPI-measurements. In future, we plan to measure the density more accurately using a femtosecond laser, in a similar fashion as Meng et al. [44]. Although the ND_3_+Ar collision cross section in this energy range does not show spectacular features, the shallow minimum around 70 cm ^−1^ predicted by theory is reproduced in the experiment.

## Conclusions

We have performed the first scattering experiment using a molecular synchrotron. Our measurements demonstrate that, by accumulating collision signal over the long time that the ammonia molecules are stored, the sensitivity is spectacularly increased. This high sensitivity has allowed us to measure the relative, total, integral cross section for ND_3_ + Ar collisions over an energy range of 40—140 cm ^−1^ with a precision of a few percent. The collision energy was tuned in three different ways: (1) by changing the temperature of the valve that releases the argon atoms, (2) by changing the velocity of the stored packets of ammonia, and (3) by choosing which part of the argon packet, dispersed during its 1.2 m traversal from the valve to the synchrotron, is probed by the ammonia molecules. These measurements give consistent results and agree with theoretical scattering calculations.

Besides the enhanced sensitivity and the relatively low energy that is obtained by using co-propagating beams, our method has a number of additional features that make it attractive: (1) By comparing packets that are simultaneously stored in the synchrotron, the measurements are independent of the ammonia intensity and immune to variations of the background pressure in the synchrotron. (2) As the probe packets interact with many argon packets, shot-to-shot fluctuations of the argon beam are averaged out. By rapidly toggling between different ammonia velocities and timings, slow drifts of the argon beam intensity are eliminated.

A detailed characterization of the argon beams was crucial for obtaining a high precision. To retrieve the collision cross section from the measured loss rates, trajectory simulations were used to evaluate the overlap of the ammonia packet with the argon beam. These simulations were also used to access the energy resolution of the measurements.

Measurements have shown that the (in-plane) alignment of the argon with respect to the synchrotron needs to be carefully considered. In the experiments presented here, the alignment was chosen in such a way that the argon beam crossed the path of the ammonia molecules at two distinct positions. This made it easy to check the validity of the simulations. A better energy resolution would be obtained, however, when the beamline would either be moved further inwards such that the collision zones are sufficiently far away from each other, or be moved outwards until only a single collision zone is left. The highest resolution would be obtained (at the cost of increasing the collision energy) by crossing the beams at right angles. This would make it possible to resolve the fine-structure on the elastic cross section due to scattering resonances predicted by Loreau et al. [43].

The collision energy is currently limited by the large difference between the velocity of the stored molecules and the velocities in the supersonic beam. Lower collision energy can be reached by using molecules from cryogenically cooled beams as collision partner [45]. As these beams typically have a much longer temporal profile, they would overlap with multiple packets. However, even in this case there will be packets that have no overlap and can serve as reference. Another strategy would be to use a larger synchrotron which would be able to store ammonia molecules at a higher velocity. Ideally, a synchrotron would be used that can store molecules directly from a supersonic beam without deceleration, resulting in higher densities. As the radius of the ring scales with the square of the forward velocity, such a ring would have to be ∼10 times larger (if the same voltages are used). Such a ring could be used to store beams both clockwise and anticlockwise, which makes it possible to perform calibration measurements at high energy. In this way it will be possible to measure collision energies from 0–2000 cm ^−1^ in the same apparatus. Note that if the velocities of the beams are more similar, the energy resolution will also be improved [36], ultimately limited to the temperature of the stored ammonia packets. Finally, collision studies with paramagnetic atoms and molecules, such as hydrogen – the most abundant atom in the universe – can be performed in a magnetic synchrotron, as described in Van der Poel et al. [46].
